# A predictive model for pain response following radiotherapy for treatment of spinal metastases

**DOI:** 10.1038/s41598-021-92363-0

**Published:** 2021-06-18

**Authors:** Kohei Wakabayashi, Yutaro Koide, Takahiro Aoyama, Hidetoshi Shimizu, Risei Miyauchi, Hiroshi Tanaka, Hiroyuki Tachibana, Katsumasa Nakamura, Takeshi Kodaira

**Affiliations:** 1 Department of Radiation Oncology, Aichi Cancer Center, 1-1 Kanokoden, Chikusa-ku, Nagoya, Japan; 2 Department of Radiation Oncology, Hamamatsu University School of Medicine, 1-20-1 Handayama, Higashi-ku, Hamamatsu, Shizuoka Japan

**Keywords:** Bone metastases, Outcomes research

## Abstract

To establish a predictive model for pain response following radiotherapy using a combination of radiomic and clinical features of spinal metastasis. This retrospective study enrolled patients with painful spine metastases who received palliative radiation therapy from 2018 to 2019. Pain response was defined using the International Consensus Criteria. The clinical and radiomic features were extracted from medical records and pre-treatment CT images. Feature selection was performed and a random forests ensemble learning method was used to build a predictive model. Area under the curve (AUC) was used as a predictive performance metric. 69 patients were enrolled with 48 patients showing a response. Random forest models built on the radiomic, clinical, and ‘combined’ features achieved an AUC of 0.824, 0.702, 0.848, respectively. The sensitivity and specificity of the combined features model were 85.4% and 76.2%, at the best diagnostic decision point. We built a pain response model in patients with spinal metastases using a combination of clinical and radiomic features. To the best of our knowledge, we are the first to examine pain response using pre-treatment CT radiomic features. Our model showed the potential to predict patients who respond to radiation therapy.

## Introduction

Radiotherapy is widely used for pain relief associated with painful bone metastases and was performed in 12.5% of all radiotherapy treatments in Japan^1^. The rate of pain relief after radiotherapy is estimated to be 60%^2^, meaning 40% of patients do not get adequate pain relief after radiation therapy. It may become possible to select the patients who do not benefit from radiotherapy, if we can predict the degree of pain relief after therapy. In a report on predictive clinical models, the World Health Organization performance status (PS), numerical rating scale (NRS), and primary tumor site were important factors for predicting pain relief^3^. However, this model did not exhibit high performance, thus better predictive models of performance are needed for clinical use.

"Radiomics” is an image analysis method involving the extraction of multiple features from medical images. By analyzing a specified area and many radiomic features from CT, MRI, PET and other images, it is possible to identify the tumor genotype and phenotype^4^. Furthermore, a database of clinical information can be created and machine learning techniques such as random forests (RF), an ensemble classifier which classifies data using decision trees and facilitates the validation and evaluation of the classification accuracy of a set of predictors^5, 6^, can be used to improve the accuracy of diagnosis and prediction of prognosis after treatment^7, 8^.

Herein, we sought to develop better diagnostic methods to determine pain response for patients with spinal metastases. We built a highly accurate model using radiomic and clinical features for predicting pain relief after radiotherapy for painful spinal metastases. Our models provide proof-of-concept for predictive tools for pain response and, with further refinement, will become an essential clinical tool for patients with bone metastases.

## Materials and methods

### Patient selection

This retrospective study was approved by our institutional review board (Aichi Cancer Center Medical Ethics Committee), with waivers for patients’ informed consents. We confirmed that all methods were performed in accordance with the relevant guidelines and regulations. Patients with painful spine metastases who received palliative radiation therapy were enrolled at the Aichi Cancer Center from 2018 to 2019. Eligible patients met the following criteria: (1) pathological diagnosis of cancer, (2) received palliative radiotherapy (i.e., a score of at least 2 on the Numerical Rating Scale (NRS) for pain) for painful spine metastasis and (3) no prior radiation to the site. Exclusion criteria were: (1) metal artifacts close to target spine, (2) palliative surgery before radiotherapy, (3) other extraspinal metastases in the radiation field and (4) no pain assessment more than 1 month after radiotherapy.

### Overall study design

**Figure 1:**
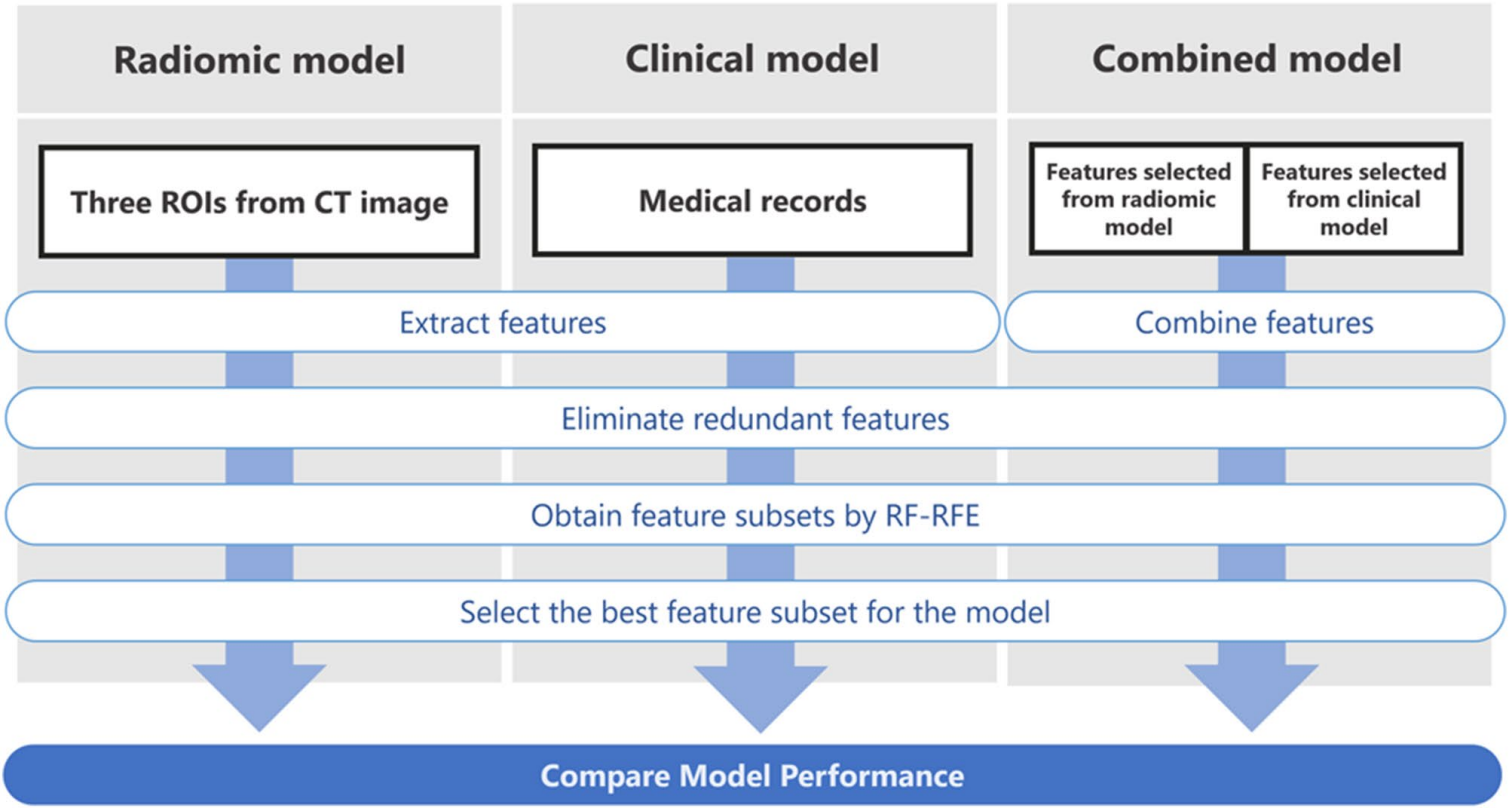
The workflow for our study of radiotherapy-induced pain response in patients with spinal metastases.

The methodological workflow is described in Fig. 1. The clinical features and radiomic features were extracted from medical records and pre-treatment CT images. Pearson’s correlation was used for the elimination of redundant features. Several feature subsets were obtained by random forests and recursive feature elimination (RF-RFE). We trained the random forests (RF) model and validated the model using leave-one-out cross-validation (LOOCV) with all the feature subsets. Finally, the feature subset with the highest area under the curve (AUC) was selected as the optimal subset for the model.

Three models including the radiomic model (radiomic feature-based model), clinical model (clinical feature-based model) and combined model (combined radiomic and clinical feature-based model) were constructed with a selected feature subset and validated using the AUC.

### Assessment items

We defined complete response or partial response based on the International Consensus Criteria for pain response^2^. Pain response was categorized as ‘pain reduction’ if there was at least a 2-point reduction in NRS at the irradiated site from the initial worst pain (without an increase in analgesic use) or an analgesic decrease of at least 25% without an increase in NRS. Patients were categorized as responders if they met the criteria for pain response at an evaluation at least 1 month after radiotherapy. Patients who did not show pain response were categorized as non-responders.

### CT image acquisition

Non-contrast enhanced CT scans for radiotherapy were used in this study. An Aquilion LB CT system (Canon Medical Systems, Tochigi, Japan) was used with the following conditions: tube voltage = 120 kV, tube current = automatic exposure control, matrix size = 512 × 512 pixels, field of view = 550 mm, and slice thickness = 3 mm.

### Segmentation of regions of interest

**Figure 2:**
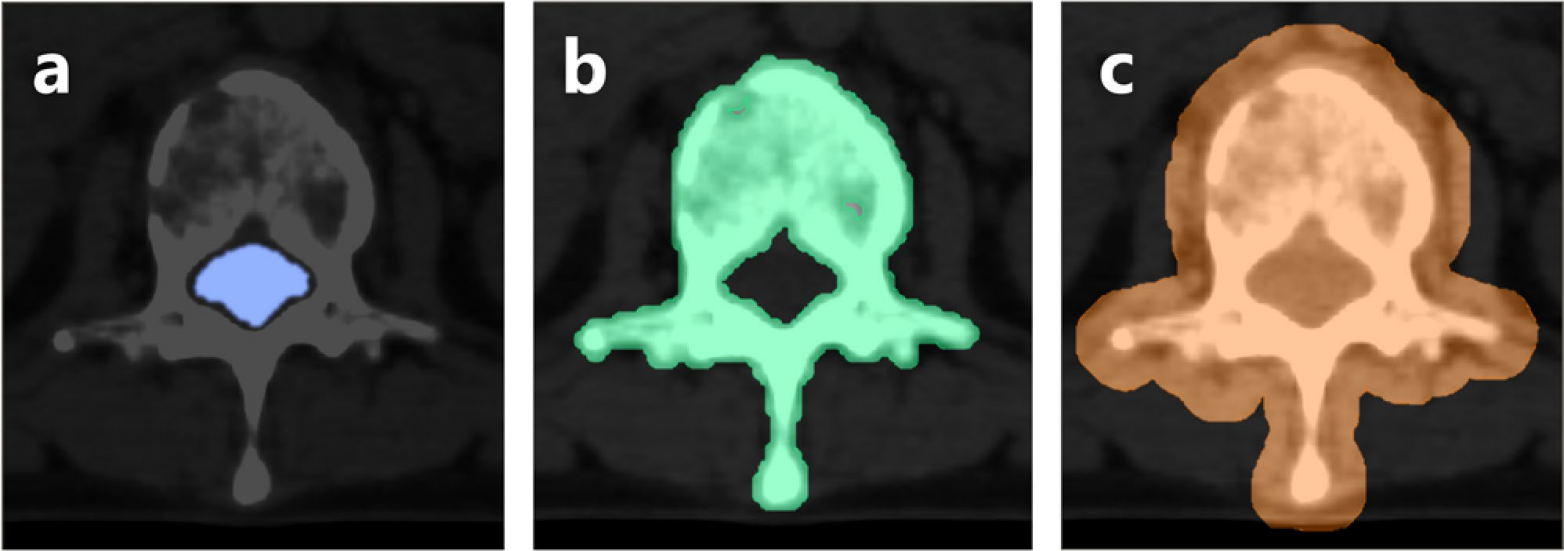
Segmentation of three regions of interest from CT images of patients with spinal metastases. (**a**) Spinal canal; (**b**) spine; (**c**) spine and surrounding tissues.

We made three regions of interest (ROI) including the spinal canal, the spine, the spine and surrounding tissues (Fig. 2). The ROI of the spinal canal was created on a RayStation (ver. 6.2, RaySearch Laboratories) using model-based segmentation (MBS), which automatically delineates organs (Fig. 2a). The ROI of the spine was delineated with MIM (ver. 7.0, MIM software Inc., Cleveland, OH) using a threshold of 150 HU and manual adjustment, such as removal of vascular calcification near the spine (Fig. 2b). The ROI of the spine and surrounding tissues was enlarged 1 cm from the ROI of the spine (Fig. 2c) in order to analyze extraspinal extending mass lesions.

In all ROIs, regions outside the treatment field were eliminated. All manual adjustments were done by one radio-oncologist with more than 2 years of experience.

### Radiomic feature extraction from CT image

The PyRadiomics Python package (version 2.2.0) was used for the extraction of radiomic features^9^. 107 quantitative radiomic features were extracted from all three ROIs including: 18 first-order statistics, 14 Gy level difference matrices, 24 Gy level co-occurrence matrix features, 16 grey-level run length matrix features, 16 Gy level size zone matrix features, 5 neighborhood gray-tone difference matrix features, and 14 shape features.

### Clinical data

The following clinical features were derived from medical records: NRS (continuous), primary tumor sites (lung, digestive, breast, head & neck or other), PS (0–1 or 2–4), gender, age (continuous), biologically effective dose (BED10) (continuous), short versions of the Leeds Assessment of Neuropathic Symptoms and Signs (S-LANSS) (0–11 or 12–24) and the spine instability neoplastic score (SINS) (0–6 or 7–12). To compare baseline categorical and continuous variables, chi-square and Mann–Whitney tests were used between the responder and non-responder groups.

### Feature selection with random forest

Features with a Pearson correlation coefficient of > 0.7 were considered dependent factors and the feature with the larger mean absolute correlation with all remaining features was eliminated. We used RF-RFE to optimize the number of features. RFE calculates the importance of the feature to determine the best subset. N features were ranked from the most to the least important (*N* is the total number of features which RFE was applied to). Then, *N* feature subsets could be obtained by selecting a different number of features. Each feature subset was fed into the RF and validated by LOOCV. Training and validation were repeated ten times and its performance could be evaluated using the score of the area under the curve (AUC). Finally, the feature subset with the highest AUC was selected as the optimal subset for the discrimination task.

In this study, all parameters of RF were default. Since small sample sizes were expected, we decided to apply our analysis using a single training cohort.

### Model building

The radiomic model was built from the radiomic features and clinical model was built from the clinical features following feature selection. In building the combined model, the selected features of the radiomic and clinical models were combined, and feature selected again. To assess the performance of each model, we used AUC, sensitivity, and specificity which were calculated by selecting the optimal feature subset.

### Statistical analysis

Statistical analysis was performed using R version 3.5.2 (The R Foundation for Statistical Computing, Vienna, Austria). The level of confidence was kept at 95% and *P* values less than 0.05 were considered significant. Receiver operating characteristic (ROC) curve analysis was performed to calculate the AUC and its corresponding 95% confidence interval.

## Results

**Figure 3:**
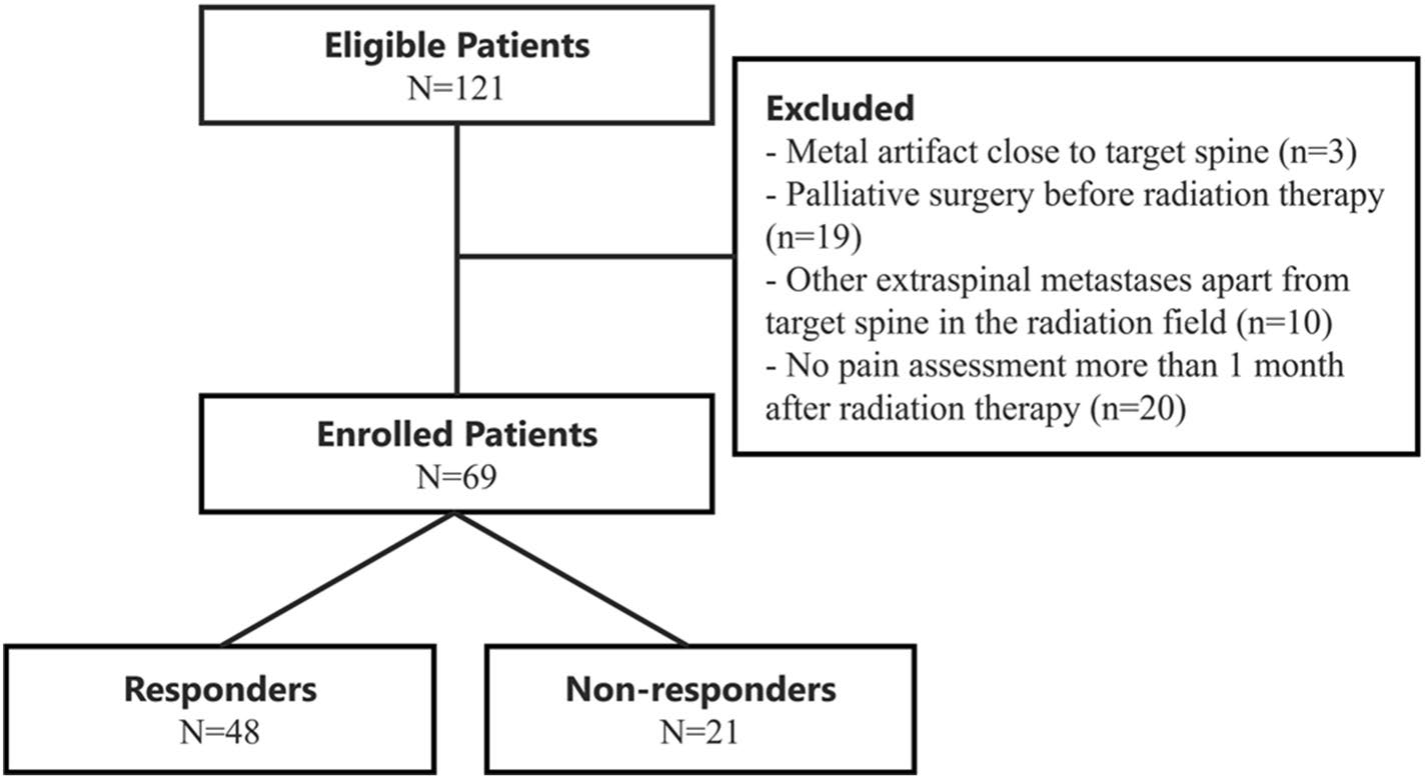
CONSORT flow diagram. Patients who received radiotherapy for painful bone metastases at the Aichi cancer center.

To better understand pain response following radiotherapy for spinal metastasis, we performed a retrospective analysis using patient clinical features and radiology-based images. 69 patients were enrolled, and their clinical features are listed in Table 1. 48 patients were classified as responders and 21 as non-responders (Fig. 3). Lung (n = 20), digestive (n = 15) and breast (n = 14) were the most common primary tumor sites. Other primary cancers included head & neck (n = 9), unknown primary (n = 4), soft tissue (n = 3), gynecological (n = 2), prostate (n = 1) and renal (n = 1). All patients received three-dimensional conformal radiotherapy delivered with photon beams generated by a linear accelerator (Synergy, TrueBeam). The results of Pearson’s correlation removed 277 redundant features from a total of 321 radiomic features. The clinical feature subset had no redundant features.

**Table 1:** Patient demographics and disease characteristics.

	Response to RT	No response to RT	p value
	(n = 48)	(n = 21)	
**Age**			0.938
Median (range)	61 (26–86)	60 (35–83)	
**Gender**			0.218
Male	25	15	
Female	23	6	
**Primary tumor site**			0.046
Lung	14	6	
Digestive	10	5	
Breast	13	1	
Head & neck	3	6	
Other	8	3	
**Radiation treatment (BED10)**			0.049
8 Gy/1fr (14.4 Gy)	6	5	
20 Gy/5fr(20.0 Gy)	29	5	
24 Gy/6fr (33.6 Gy)	9	8	
30 Gy/10fr (39.0 Gy)	4	3	
**WHO performance status**			1
0–1	44	19	
2–3	4	2	
4	0	0	
**Numeric rating scale**			0.102
Median (range)	5 (0–10)	7 (0–10)	
**S-LANSS**			0.22
0–11	40	14	
12–24	8	7	
**SINS**			0.528
0–6	13	8	
7–18	35	13

**Figure 4:**
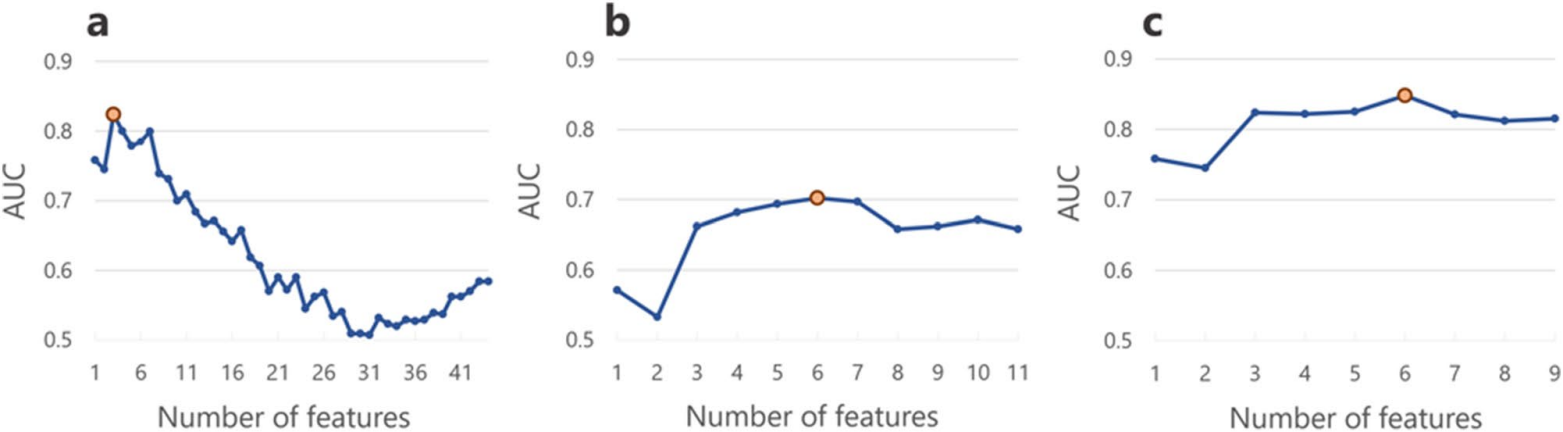
A comparison of feature subsets for predictive models of pain response using the area under receiver operating characteristic curve. (**a**) Radiomic model; (**b**) clinical model; (**c**) combined model. The optimal number (orange dot) was determined from the highest AUC. The clinical, radiomic and combined feature subsets with the highest AUC contained 6, 3 and 6 features, respectively.

**Figure 5:**
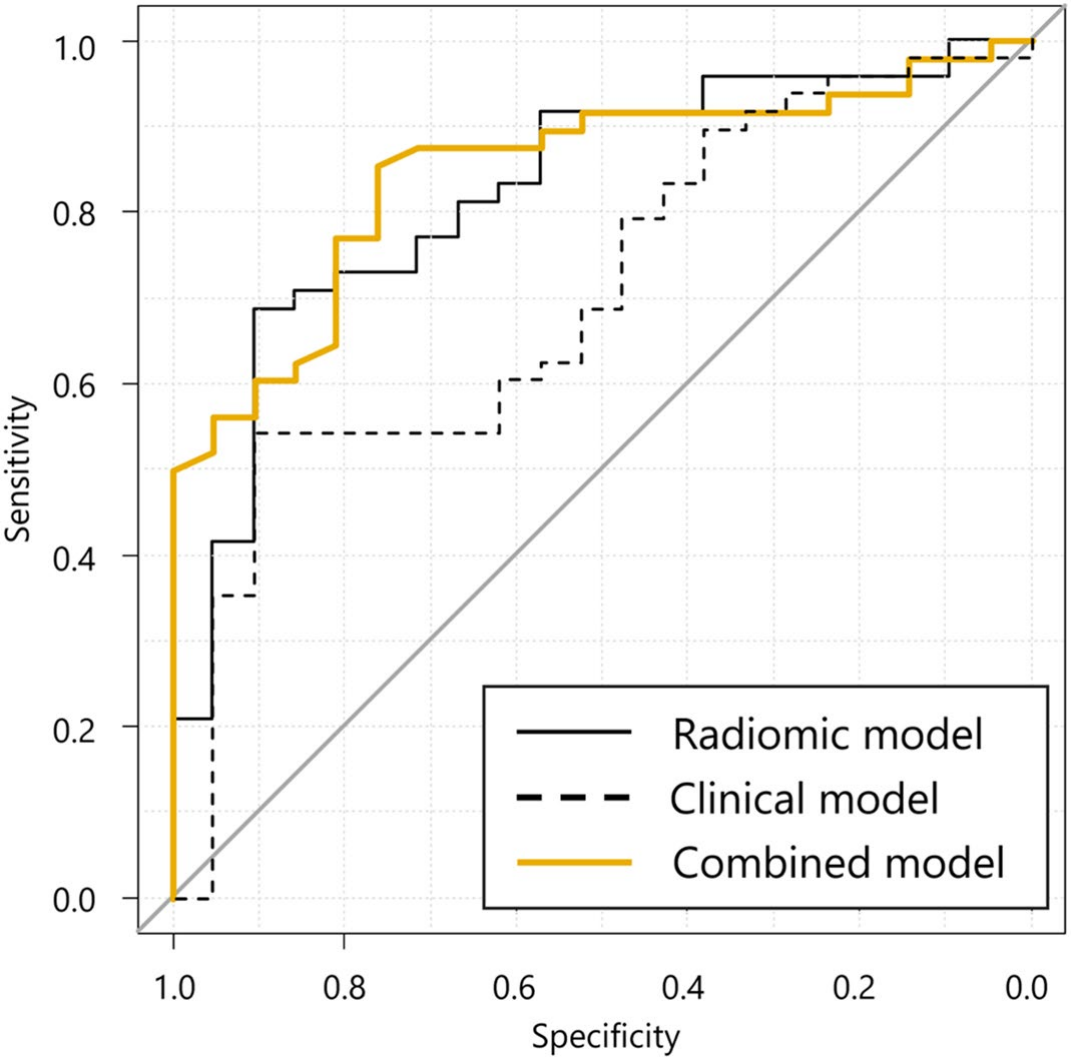
Receiver operating characteristic (ROC) curves of predictive models for pain response. The combined model had a higher area under curve (0.848) than the other parameters and was not significantly better than radiomic model (*p* = 0.599), however; it was significantly greater than the clinical model (*p* = 0.044).

Based upon the feature subset, random forests and recursive feature elimination (RF-RFE) was performed. The feature selection process is depicted in Fig. 4a,b. The clinical feature subset and radiomic feature subset with the highest AUC contained 6 and 3 features, respectively. To construct a combined model, RF-RFE was performed and the combined model with the highest AUC (Fig. 4c) contained 6 feature subsets (3 clinical and 3 radiomic features). The selected features in each model are listed in Table 2. The ROC curves and AUC are shown in Fig. 5 and Table 3, respectively. The best model was the combined model, with an AUC of 0.848 and accuracy of 82.6%. The combined model was not significantly better than radiomic model (*p* = 0.599) and the radiomic model was not significantly better than the clinical model (*p* = 0.208), however; it was significantly greater than the clinical model (*p* = 0.044). To this end, we have developed a predictive model which will aid in the determination of patient outcome when treating spinal metastases with radiotherapy.

**Table 2:** The model features after feature selection.

Model	Feature		
Radiomic	S_LeastAxisLength	S_MCC	SS_Idmn
Clinical	age	S-LANSS	BED10
	NRS	PTS_Digestive	PTS_Head & neck
Combined	S_LeastAxisLength	S_MCC	SS_Idmn
	age	BED10	NRS

*S_LeastAxisLength* LeastAxisLength from an ROI of spine, *S_MCC* MCC from an ROI of spine, *SS_Idmn* Idmn from an ROI of spine and surrounding tissues, *PTS_Digestive* primary tumor site is digestive, *PTS_Head & neck* primary tumor site is Head & neck.

**Table 3:** Area under receiver operating characteristic curve, sensitivity, specificity and accuracy of the predictive models for pain response.

Model	AUC	95% CI	Sensitivity (%)	Specificity (%)	Accuracy (%)
Radiomic	0.824	0.718–0.931	70.80	85.70	73.90
Clinical	0.702	0.568–0.840	54.20	90.50	65.20
Combined	0.848	0.755–0.940	85.40	76.20	82.60

## Discussion

Incomplete patient response to radiotherapy for painful spinal metastases greatly hinders quality of life. Using a combination of clinical features and radiomics, we sought to establish a predictive model for patient pain response to radiotherapy treatment. Our study found that a model combining pre-treatment CT based radiomic features and clinical features showed good predictive scores for pain relief after palliative radiation in patients with painful spinal metastases compared to a clinical feature-based model.

In our study, age, NRS, and BED10 were determined as important features using our combined model. Previous studies have reported that pain response is associated with age and NRS^10, 11^. Other features which have been reported as affecting pain response after radiotherapy are gender, performance status, primary tumor site, neuropathic pain, Bilsky grade, prior radiation at the site of interest, and sleep disturbance^7, 11–14^. In this study, we evaluated NRS and S-LANSS as pain indices because they are widely used, and were recorded in all patients. SINS, which we also evaluated, is an indicator of spinal instability and we referred it to when consulting with the spine surgery department or at multidisciplinary conferences. The AUC in previous studies that modeled pain response from clinical features was 0.63^3^. All of the models created in our study yielded better AUCs than previous reports, suggesting our refined methods were more successful in generating accurate models.

Radiomics is a non-invasive way to obtain high-dimensional, mineable and quantitative data from medical images such as CT scans. CT scans are routinely taken for diagnostic purposes. CT scan are also taken during and after treatment to check for changes in lesion size, shape, and characterization. Compared to blood tests and biopsies, radiomics does not require specialized equipment, making it an accessible diagnostic option. The goal of radiomics is to improve diagnosis and prediction in clinical practice.

Previous studies that did not use radiomic features have investigated medical images to assess the response of bone metastases after therapy^15–18^. In contrast, radiomics is able to extract more information from the ROI, such as intensity and shape, which can be combined to create a model with high predictive and diagnostic capabilities^19–21^. There have been several studies examining bone metastases utilizing radiomics^22–25^. To the best of our knowledge, we are the first to examine pain response using pre-treatment CT radiomic features.

Pain from bone metastases is a common symptom with many patients exhibiting debilitating pain^26^. Radiotherapy is an important treatment for pain relief from bone metastases, however; the inability to move for several minutes can cause pain. If the patient moves, there is a risk of injury. In the case of palliative irradiation of bone metastases, the radiation dose is low, but it is not without the potential for adverse events. Considering these aforementioned risks associated with therapy, it is important to identify patients who have a higher likelihood to respond to treatment. If the study can identify patients who are not expected to respond, it will be possible to choose alternative treatments such as higher-dose radiotherapy, early surgical intervention, switching opioid regimens or systemic therapy.

Some limitations from our study should be noted. The number of cases was small. 52 patients were excluded from our initial population owing to our exclusion criteria. The anthropogenic changes in spinal structures and metal artifacts due to surgery, which were included in the exclusion criteria, were thought to influence some radiomic features and this was expected to lead to a decrease in prediction accuracy. In addition, we excluded re-irradiated cases. The exclusion of re-irradiated cases was done to create an accurate model with a more homogeneous group of cases.

Due to the limited sample size, the evaluation of our model was measured based on the training cohort, and not the test cohort. Only examining the training cohort caused overfitting in feature selection and training on RF. We avoided overfitting by using LOOCV and not using hyperparameter optimization. Second, segmentation may also need to be investigated. In the present study, we used a semi-automatic segmentation approach for the spine and surrounding structures using CT number. This allows us to reduce the differences in manual segmentation between images. In some cases, it is possible that most of the volume of the ROI may have been normal structures. It is preferable to evaluate the radiomic features of the actual bone lesion, but some patients did not have an MRI which is required to accurately delineate bone lesions. We decided to include patients who did not have an MRI in order not to reduce the number of enrolled patients and abandoned the evaluation of the ROI based on segmentation of the actual bone lesion. We also abandoned the analysis of MRI radiomic features which were used to analyze bone metastatic lesions.

In the future to address the limitations of our study, we will conduct a multicenter observational study, which will expand the sample size and prepare the external validation set to test the prediction performance and generalization capacity of our model. Future studies will be needed to consider re-evaluating segmentation settings and usage of MRI images.

In conclusion, we created a model combining clinical and radiomic features to predict patients who respond to radiotherapy for spinal metastases. We hope that our model, and subsequent refinements, will inform on clinical decisions for patients with bone metastases and improve their quality of life.

## Abbreviations

BED: Biologically effective dose
LOOCV: Leave-one-out cross-validation
MBS: Model-based segmentation
NRS: Numerical rating scale
PS: Performance status
RF: Random forest
RFE: Recursive feature elimination
SINS: Spine instability neoplastic score
S-LANSS: Short versions of the Leeds Assessment of Neuropathic Symptoms and Signs
